# Ovarian Carcinoma Presenting Mucoepidermoid Carcinoma-Like Features in Association With Seromucinous Borderline Tumor: A Real Seromucinous Carcinoma?

**DOI:** 10.7759/cureus.114557

**Published:** 2026-08-15

**Authors:** Kentaro Ishida, Ayaka Nakamura, Atsuko Taga, Tomoyuki Shirase, Yoshiki Mikami, Kohei Fujita

**Affiliations:** 1 Department of Obstetrics and Gynecology, Japanese Red Cross Otsu Hospital, Otsu, JPN; 2 Department of Obstetrics and Gynecology, Kyoto University Graduate School of Medicine, Kyoto, JPN; 3 Department of Pathology and Diagnosis, Japanese Red Cross Otsu Hospital, Otsu, JPN; 4 Department of Surgical Pathology, Kumamoto University, Kumamoto, JPN

**Keywords:** mucoepidermoid carcinoma, ovarian cancer, p40, seromucinous borderline tumor, seromucinous carcinoma, who classification

## Abstract

Ovarian seromucinous carcinoma is generally classified within the spectrum of endometrioid carcinoma. We report a rare ovarian carcinoma with mucoepidermoid carcinoma-like features arising in direct association with a seromucinous borderline tumor. A 56-year-old postmenopausal female presented with a 10-cm pelvic multilocular cystic tumor and markedly elevated carbohydrate antigen 19-9. Histopathological examination showed a seromucinous borderline tumor with transition to invasive carcinoma composed of mucinous epithelial cells and p40-positive intermediate-like cells. The tumor was diffusely positive for PAX8 and negative for cytokeratin 20, supporting Mullerian differentiation. The p40-positive cell population persisted in the borderline, invasive, and para-aortic lymph node metastatic components. Comprehensive genomic profiling identified KRAS p.G12D without CTNNB1, PTEN, or ARID1A mutations. The patient developed platinum-resistant recurrence and died 11 months after diagnosis. This case is compatible with a potential endometriosis-independent pathway from seromucinous borderline tumor to an aggressive mucoepidermoid-like carcinoma.

## Introduction

Ovarian seromucinous tumors are epithelial neoplasms composed of serous and mucinous, usually endocervical-type, cells and are frequently accompanied by squamous, transitional, and clear cell differentiation [1-3]. The 2014 World Health Organization (WHO) classification introduced seromucinous carcinoma as a distinct malignant entity [1]. However, the category remained controversial because of poor diagnostic reproducibility and overlap with endometrioid carcinoma in both morphology and molecular alterations [4,5]. The 2020 WHO classification therefore removed seromucinous carcinoma as a separate category and recommended that most such tumors be classified as endometrioid carcinoma [4].

Despite this reclassification, seromucinous borderline tumor (SMBT) remains a recognized precursor lesion, and its biology may not be identical to that of endometrioid borderline tumor. A characteristic feature of SMBT is the presence of subepithelial cuboidal or eosinophilic cells, sometimes described as reserve-like or intermediate-like cells, which can express p63 or p40 [6]. Direct progression of this cell population into invasive carcinoma has rarely been documented.

We report a case of ovarian carcinoma with mucoepidermoid carcinoma-like features arising in association with SMBT. The tumor maintained a p40-positive intermediate-like cell population from the borderline component to invasive carcinoma and lymph node metastasis, without endometriosis or a conventional endometrioid carcinoma component. The case highlights a possible distinct pathway of Mullerian carcinogenesis.

## Case presentation

A 56-year-old postmenopausal female, gravida 2 para 2, presented to a local physician with lower abdominal pain. Serum tumor marker levels were elevated, with carbohydrate antigen 19-9 (CA19-9) at 262 U/mL (reference range, <37 U/mL) and carcinoembryonic antigen (CEA) at 7.6 ng/mL (reference range, <5.0 ng/mL). Transvaginal ultrasonography showed a 10-cm multicystic pelvic mass, and she was referred to our hospital for further evaluation.

**Figure 1:**
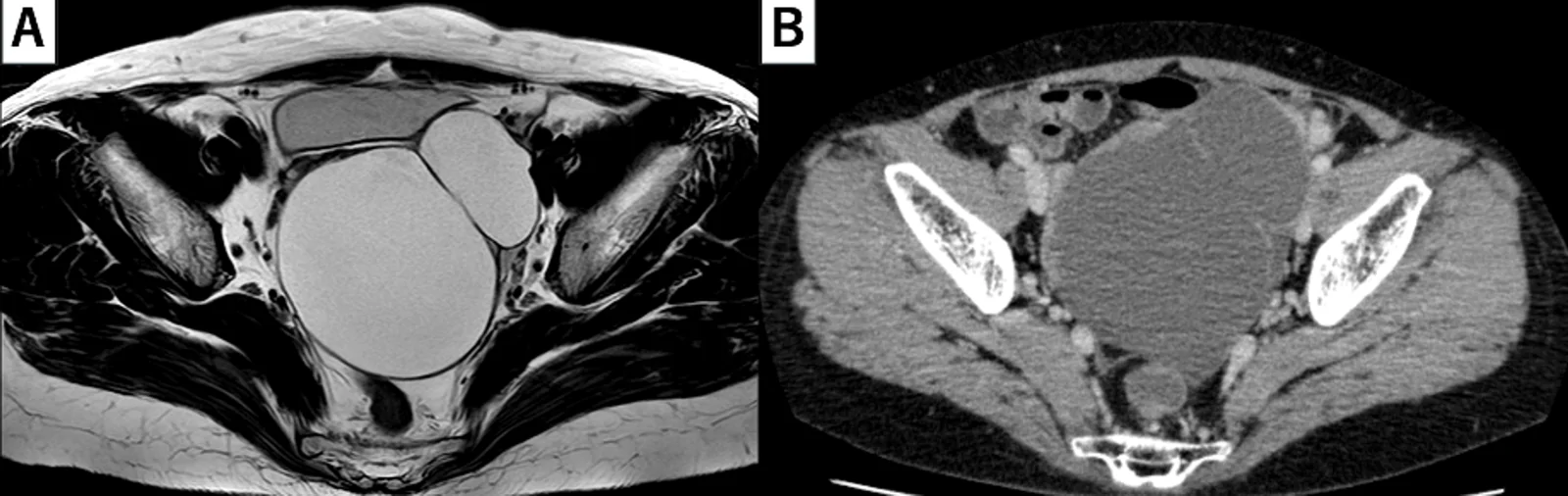
Radiologic findings. (A) Axial T2-weighted magnetic resonance imaging showed a large multilocular cystic tumor in the pelvis. (B) Contrast-enhanced computed tomography revealed no enhancement of the cyst wall or internal septa; there were no findings suggestive of malignancy.

Her medical and family histories were unremarkable. CA19-9 was markedly elevated at 1,273.9 U/mL at the time of referral, whereas CEA and cancer antigen 125 (CA125) were within normal limits. Pelvic magnetic resonance imaging (MRI) demonstrated a 9 × 8 cm multilocular cystic tumor arising from the left ovary, with variable intracystic signal intensity but no enhancement of solid components or septa on post-contrast images (Figure 1A). Contrast-enhanced computed tomography (CT) of the chest, abdomen, and pelvis showed no enhancement of the cyst wall or septa, and no distant metastasis (Figure 1B).

**Figure 2:**
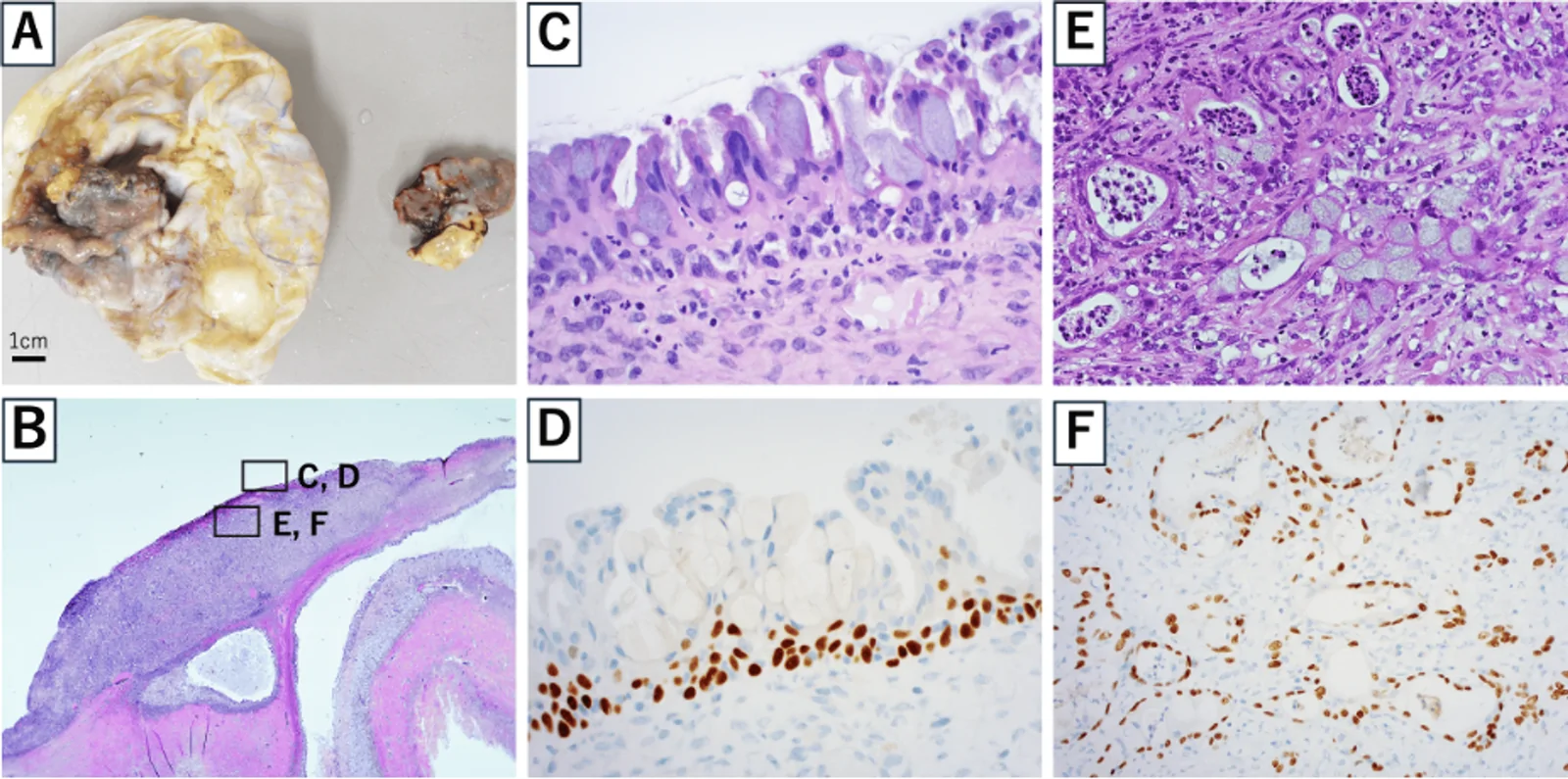
Histopathological and immunohistochemical findings. (A) A 9-cm cyst containing brownish mucinous fluid was present with an inner surface that was mostly smooth apart from focal papillary projections. (B) Low-power view of the seromucinous borderline tumor component within the cyst wall (hematoxylin and eosin, original magnification ×10). (C) At higher magnification, the cysts are lined by endocervical-type mucinous cells underlaid by eosinophilic intermediate-like cells (hematoxylin and eosin, ×100). (D) The intermediate-like cells show strong, diffuse nuclear p40 positivity (p40 immunohistochemistry, x100). (E) Irregular glandular structures infiltrate the ovarian stroma while retaining the two-cell pattern (hematoxylin and eosin, x200). (F) Intermediate-like cells within the invasive glands maintain strong p40 expression, highlighting the bilayered architecture (p40 immunohistochemistry, x200).

As malignancy could not be excluded, exploratory laparotomy was performed one month after referral. Preoperatively, CA19-9 had risen further to 19,694.5 U/mL. The cystic tumor was found to have ruptured, and left salpingo-oophorectomy was performed. Intraoperative frozen-section analysis was inconclusive for malignancy. The uterus and right ovary appeared normal, and no gross endometriosis or peritoneal dissemination was observed. Ascitic fluid cytology was negative for malignant cells. Gross examination revealed a 9-cm cyst containing brownish mucinous fluid, with an inner surface that was mostly smooth apart from focal papillary projections (Figure 2A). Histological examination showed an SMBT composed of endocervical-type mucinous cells with one to several subjacent layers of eosinophilic cuboidal cells resembling the intermediate cells of mucoepidermoid carcinoma (Figures 2B, 2C). Immunohistochemistry showed diffuse PAX8 positivity and cytokeratin 20 negativity, consistent with Mullerian origin. The intermediate-like cells showed strong, diffuse nuclear p40 positivity (Figure 2D). In some areas, bilayer glands containing both mucinous and p40-positive intermediate-like cells irregularly invaded the ovarian stroma (Figure 2E). The invasive glands retained the same two-cell pattern, with p40 highlighting the intermediate-like cells (Figure 2F). The tumor penetrated the ovarian capsule.

After the definitive diagnosis, the patient underwent complete surgical staging, including total hysterectomy, right salpingo-oophorectomy, omentectomy, and pelvic and para-aortic lymphadenectomy. Metastatic carcinoma was identified in one para-aortic lymph node. The metastatic deposit also contained p40-positive intermediate-like cells, confirming persistence of the phenotype in the metastatic component. The final diagnosis was ovarian carcinoma with mucoepidermoid features arising in an SMBT, International Federation of Gynecology and Obstetrics (FIGO) 2014 stage IIIA1(ii) [7].

The patient received six cycles of adjuvant paclitaxel and carboplatin. However, platinum-resistant recurrence occurred one month after completion of chemotherapy. Comprehensive genomic profiling was performed on primary tumor tissue using the GenMineTOP cancer genomic profiling system (Konica Minolta REALM, Inc., Tokyo, Japan), a paired tumor-normal, dual DNA/RNA next-generation sequencing assay that detects base substitutions, insertions/deletions, and copy-number alterations in 737 genes and gene fusions in 455 genes [8]. The analysis identified KRAS p.G12D as the dominant pathogenic alteration, with additional CTR9, EP300, and TERT coding-region variants (Table 1). No CTNNB1, PTEN, ARID1A, or TERT promoter mutation was detected, and no actionable therapeutic target was identified. The tumor mutational burden was low at 2.1 mutations/Mb, and no reportable copy-number alteration was identified. RNA analysis detected no fusion transcript or exon-skipping event. RNA expression profiling showed no clearly actionable expression outlier; although CDK4 and MYC expression levels were relatively high, these findings were not accompanied by copy-number alterations. No reportable pathogenic germline variant was detected. No guideline-recommended therapeutic target was identified. The patient was transitioned to palliative care and died of disease approximately 11 months after the initial diagnosis.

**Table 1:** Genomic alterations detected by comprehensive genomic profiling. Variants were detected using the GenMineTOP cancer genomic profiling system (paired tumor-normal DNA/RNA sequencing). Allele frequency is shown as the number of variant reads over the total read depth, with the variant allele fraction in parentheses. ClinVar and COSMIC classifications are those recorded at the time of analysis; COSMIC values in parentheses indicate the FATHMM pathogenicity score. All variants were somatic; no reportable pathogenic germline variant was detected. CDS: coding DNA sequence; COSMIC: Catalogue of Somatic Mutations in Cancer; FATHMM: Functional Analysis through Hidden Markov Models.

Gene	Chromosomal location	CDS change	Protein change	Allele frequency	ClinVar	COSMIC
CTR9	11p15.4	c.43G>A	p.E15K	123/1,420 (8.7%)	Likely pathogenic	Not listed
EP300	22q13.2	c.4241A>G	p.Y1414C	51/1,011 (5.0%)	Not listed	Pathogenic (0.99)
KRAS	12p12.1	c.35G>A	p.G12D	155/1,136 (13.6%)	Pathogenic	Pathogenic (0.98)
TERT	5p15.33	c.2449G>A	p.A817T	73/1,387 (5.3%)	Conflicting interpretations	Neutral (0.01)

## Discussion

This case presents a rare ovarian carcinoma with mucoepidermoid features originating directly from an SMBT, challenging the current WHO classification system. It is notable for its aggressive clinical course, its distinctive p40-positive biphasic immunoprofile, and its KRAS-mutant, PTEN/ARID1A/CTNNB1-wildtype molecular signature.

Although this tumor must currently be regarded as an endometrioid carcinoma with mucinous differentiation under the 2020 WHO classification [4,5], several features challenge this simple categorization. The reclassification of seromucinous carcinoma as a subtype of endometrioid carcinoma was based on three principal arguments: limited diagnostic reproducibility, frequent association with endometriosis, and shared molecular alterations such as KRAS and PTEN mutations [4,5]. However, recent evidence suggests that this conceptual unification may obscure biologically distinct entities. Tomita et al. recently described a seromucinous borderline tumor with a predominant serous component and proposed that a subset of such tumors may be better regarded as a serous borderline tumor with mucinous differentiation, the borderline counterpart of low-grade serous carcinoma with mucinous differentiation, an entity not addressed by the current WHO classification [9]. In parallel, Niu et al. demonstrated [10], through comprehensive next-generation sequencing of 11 endometrioid borderline tumors (EBTs) and 10 SMBTs, that these two entities possess fundamentally divergent molecular landscapes: EBTs are dominated by WNT/beta-catenin signaling with frequent CTNNB1 mutations (73%), whereas SMBTs lack CTNNB1 mutations and instead show RAS-MEK-ERK pathway alterations driven by KRAS (60%) and BRAF (30%) mutations. These findings indicate that, at least at the borderline stage, SMBTs and EBTs are molecularly distinct, raising the question of whether their malignant counterparts should be unified under a single endometrioid umbrella.

In our case, the absence of a conventional endometrioid carcinoma component, the absence of associated endometriosis, and the intimate spatial and morphological continuity between the borderline and carcinomatous elements collectively argue that this neoplasm may qualify as a bona fide seromucinous carcinoma. The morphology of the invasive component, with its intermediate cells, squamoid features, and mucin production, closely mimics mucoepidermoid carcinoma (MEC), a tumor characteristic of the salivary glands. The defining feature in our case was the two-cell pattern, highlighted by p40-positive cells at the periphery of the tumor nests that was strictly maintained from the borderline component through the invasive carcinoma and into the lymph node metastasis.

A critical finding is the presence of these p40-positive cells in both the SMBT component and the invasive carcinoma, suggesting a direct histogenetic link. This observation aligns with the findings of Hamada et al., who demonstrated subepithelial cuboidal cells positive for p63 in nine of 10 SMBTs and likened them to uterine cervical reserve cells [6]; p40 is the antibody that recognizes the delta-Np63 isoform of p63, and the two markers highlight the same reserve-like population. They proposed that this progenitor-like population may differentiate into various Müllerian cell types. Our case provides morphological and immunohistochemical support for this hypothesis: these p40/p63-positive cells not only exist within the SMBT but persist as an integral component of the invasive tumor while retaining their characteristic basal/intermediate position, mirroring the biphasic architecture of salivary gland MEC. To our knowledge, invasion and lymph node metastasis by a p40-positive intermediate cell population within an SMBT-derived carcinoma has rarely been documented, providing morphological evidence that the proposed reserve-like cells of SMBT may possess neoplastic potential.

The molecular findings are compatible with this interpretation. Comprehensive genomic profiling identified a KRAS p.G12D mutation (allele frequency 13.6%; ClinVar pathogenic, COSMIC pathogenic) as the dominant driver, without concurrent CTNNB1, PTEN, or ARID1A mutations. This profile is compatible with the recently characterized molecular signature of SMBT, in which KRAS mutations constitute the predominant driver event and CTNNB1 mutations are characteristically absent [10,11]. The absence of PTEN and ARID1A mutations, alterations recurrently observed in endometrioid carcinoma [5,10], is likewise compatible with an SMBT-derived origin. This pattern also diverges from the typical profile of endometriosis-associated ovarian carcinoma, in which ARID1A loss is a hallmark event, consistent with the absence of endometriosis in the surrounding ovarian tissue. No TERT promoter mutation was detected; an additional TERT coding-region variant (p.A817T) was identified but is predicted to be functionally neutral (COSMIC score 0.01) and was not considered a driver.

Two additional variants of uncertain significance (CTR9 and EP300) were also detected. Given the low tumor cellularity (approximately 20%), a fully clonal heterozygous mutation would yield an expected variant allele frequency (VAF) of approximately 10%. Therefore, the CTR9 p.E15K variant (VAF 8.7%), similar to the KRAS mutation (VAF 13.6%), likely represents a clonal event rather than a minor subpopulation. CTR9 is involved in transcriptional regulation, though its role in ovarian neoplasia is uncharacterized. Conversely, the EP300 p.Y1414C variant (VAF 5.0%) suggests a major subclonal population. Because EP300 is implicated in epithelial differentiation, its subclonal emergence might potentially relate to the distinct biphasic morphology of this tumor, although this remains speculative.

The salivary gland analogy raises the question of whether SMBT-derived, MEC-like carcinomas might share the CRTC1-MAML2 rearrangement characteristic of salivary gland MEC and of extrasalivary MEC at other anatomic sites [12]. The RNA-based fusion assay did not detect a CRTC1-MAML2 fusion. Given the low tumor cellularity (approximately 20%), however, a MAML2 rearrangement cannot be entirely excluded, and dedicated confirmatory testing, such as reverse transcription-polymerase chain reaction (RT-PCR) or fluorescence in situ hybridization (FISH), was not performed. This remains a question for future work.

Clinically, the tumor behaved aggressively, with para-aortic lymph node metastasis, rapid platinum-resistant recurrence, and death within one year. This course contrasts with the generally indolent behavior expected of low-grade endometrioid carcinomas and of SMBTs [2,3], and also with the typically low-grade behavior of salivary MEC. Takeuchi et al. recently reported SMBTs coexisting with carcinoma in endometriotic cysts and likewise emphasized that the carcinomatous component, once it emerges from SMBT, may pursue a more aggressive course than the SMBT itself [13]. Our case extends this observation to an endometriosis-independent setting and suggests that a MEC-like morphology with a persistent p40-positive intermediate-cell population may itself be a marker of aggressive behavior and may warrant further study.

## Conclusions

We describe a rare ovarian carcinoma with mucoepidermoid carcinoma-like features arising directly from an SMBT, characterized by persistence of a p40-positive intermediate-cell population from the borderline lesion through invasion into lymph node metastasis, a KRAS-driven molecular signature compatible with an SMBT-derived origin, and an aggressive course with platinum-resistant recurrence and death within one year. These features raise the possibility that a subset of SMBT-associated carcinomas may not be fully accommodated by the current endometrioid category and warrant further characterization.
